# Comparison between dual energy X-ray absorptiometry and calcaneal quantitative ultrasound for determining bone mineral density in children living with HIV in uganda: A cross- sectional study

**DOI:** 10.1186/s12887-025-05881-5

**Published:** 2025-07-04

**Authors:** Eva Natukunda, Alexander J. Szubert, Alasdair Bamford, Katja Doerholt, Diana M. Gibb, Centurio Wandera, Aidah Nakalyango, Anna Griffiths, Lara Monkiewicz, Joan J. Nangiya, Esther Nambi, Victor Musiime, Phillipa Musoke

**Affiliations:** 1 Joint Clinical Research Centre, Kampala, Uganda; 2 Medical Research Council Clinical Trials Unit, University College London, London, England; 3 Makerere University College of Health Sciences, Kampala, Uganda

**Keywords:** HIV, Bone mineral density, Quantitative ultra sound, DXA scan, Children

## Abstract

**Background:**

The aim of this study was to compare quantitative ultrasound (QUS) and dual energy X-ray absorptiometry (DXA) for determining bone mineral density (BMD) among children living with HIV (CLWH) who were switching to second-line antiretroviral therapy (ART).

**Methods:**

We conducted a cross-sectional study among CLWH as a sub-study of the CHAPAS-4 trial. Total body less head (TBLH) BMD and lumbar spine (LS) BMD were determined by DXA while the sound of speed (SOS), broad band ultrasound attenuation (BUA) and bone quality index (BQI) were determined by QUS. We evaluated the correlation between the DXA and QUS measurements using spearman correlation coefficient.

**Results:**

A total of 167 children were enrolled; the median age was 9.4 (interquartile range = 6.0–12.0) years. Eighty-five (50.9%) were male. The median weight- for- age Z- score (IQR) was − 1.29(-2.16, -0.49) and height for age Z-score was − 1.03(-1.56, 0.01). SOS was weakly correlated with TBLH BMD *R* = 0.35, *P* < 0.001), lumbar spine bone apparent density (LSBMAD) (*R* = 0.19, *P* = 0.01) and LS bone mineral content (BMC) (*R* = 0.31, *P* < 0.001). BUA was moderately correlated with TBLH BMD (*R* = 0.50, *P* < 0.001), BMC (*R* = 0.47, *P* = 0.001), and LS BMC (*R* = 0.43, *P* < 0.001) but weakly correlated with LSBMAD (*R* = 0.28, *P* < 0.001). BQI was moderately correlated with TBLH BMC (*R* = 0.46, *P* < 0.001), TBLH BMD (*R* = 0.46, *P* < 0.001) and LSBMC (*R* = 0.41, *P* < 0.001). There was weak correlation between LSBMAD and BUA (*R* = 0.28, *P* < 0.001) BQI (*R* = 0.29, *P* < 0.001). QUS Z-score was weakly correlated with TBLH BMD and (*R* = 0.30, *P* < 0.01) and no correlation with LSBMAD (*R* = 0.07, *P* = 0.35).

**Conclusion:**

In CLWH, there was moderate correlation observed for TBLH measurements when comparing DXA to QUS, and weak correlation was found between LSBMAD and QUS measurements. There was a moderate correlation detected between the LSBMC and QUS.QUS may not be an appropriate substitute for DXA scan.

## Introduction

Globally, there are 39 million people living with HIV (PLWH) with 1.3 million new infections. 1.4 million are children living with HIV(CLWH) [1]. 90% of the children live in the sub-Saharan Africa. Due to the scale up of antiretroviral therapy, children are now surviving much longer into adulthood. However, longstanding HIV infection is associated with comorbidities that may result from antiretroviral drug use or HIV per se. Poor bone health has been identified as one of the comorbidities in PLWH, and this condition may be exacerbated by malnutrition [2, 3]. Over 80% of peak bone mass is acquired during childhood and puberty, which is particularly relevant for children living in resource-limited settings (RLS) [4, 5].

Low bone mineral density (BMD) is more common and the risk of fractures is greater among PLWH than negative individuals [6, 7], with a reported prevalence ranging from 4 to 32% among CLWH. Risk factors include the use of ART especially tenofovir disoproxil fumarate, physical inactivity, low body mass index and malnutrition [2, 8–10]. Dual energy X-ray absorptiometry (DXA) remains the gold standard but it is expensive, requires trained personnel and is not widely available in most RLS where the majority of CLWH reside [11]. Further to this, DXA BMD is a two-dimensional measurement that tends to underestimate BMD for smaller bones and does not assess bone macro and microarchitecture that contribute to bone strength [12, 13].

The quantitative ultra sound (QUS) is a low cost, portable, easy to use and radiation free alternative tool for assessing BMD [14]. It measures the speed of sound (SOS), and its attenuation, the broadband ultrasound attenuation (BUA) and a third parameter, the bone quality index (BQI) which measure bone quality and structure. This low-cost tool may be used for early detection of bone loss in RLS. The calcaneal bone is recommended for determining BMD by the International Society of Clinical Densitometry (ISCD) because it is rich in trabecular bone with high turnover, has flat surfaces where the transducers can easily be placed, is accessible, and has been widely researched [15, 16].

Despite its advantages, the QUS has limitations as well like lack of standardization across the devices, inaccuracies by generating abnormally high values for children with small feet that are less than 19 cm in length [17, 18]. The anatomical site for assessment can vary among patients, requiring the use of foot supports of different sizes to position the heel introducing variations in the readings [14].

Studies comparing DXA and QUS have been largely performed in resource rich settings and have shown inconsistent results. A study among CLWH in South Africa revealed a moderate to weak correlation between DXA and QUS values [19]. In contrast, Srichan et al. reported a poor correlation in healthy Thai children when the correlation between DXA and the SOS was evaluated [20].

Few studies have evaluated the role of QUS in determining bone mineral density among CLWH with viral non suppression especially in RLS [21]. https://www.natap.org/2014/CROI/croi_67.htm.

In this study we address the gap by evaluating how QUS can be compared with DXA scan for determining BMD among CLWH who are switching to second-line ART in a resource limited setting. Findings may support the integration of QUS as a practical screening tool where DXA is unavailable to detect low BMD.

## Materials and methods

We conducted a cross-sectional study from January 2019 to March 2021 at the Joint Clinical Research Centre (JCRC) in Kampala Uganda. JCRC is an HIV care, treatment and research Centre. We used baseline data from the toxicity sub-study of the CHAPAS 4 trial (ISRCTN 22964075) a multicenter study that evaluated alternative second-line antiretroviral therapy among 919 CLWH in Uganda, Zambia and Zimbabwe.

The desired sample size was calculated based on the expected correlation coefficient (*r* = 0.3) according to Cohen’s guidelines [22] https://www.utstat.toronto.edu/brunner/oldclass/378f16/readings/CohenPower.pdf given the inconsistent and limited literature. Using Fisher’s transformation formula [23] *n*=(C(r)Zα​+Zβ​​)^2^ +3, Zα​ = Z-score for the significance level, Zβ​ = Z-score for desired power 80% power, C(r) = Fisher’s z-transformed correlation coefficient. We adjusted the alpha level by twenty-four the anticipated multiple comparisons of the DXA and QUS scan variables, a minimum sample size of 160 participants was required to detect significant correlation.

A subset of 167 children aged 3–15 years from the CHAPAS 4 trial were enrolled at JCRC for this sub-study. We included children transitioning to second line ART and had baseline BMD and QUS measurements available.

We excluded - pregnant participants and children with deformities involving the lower limbs. The participants’ BMD and bone quality were measured in parallel using DXA scan and calcaneal QUS (SONOST 3000) respectively.

### Measurements

A case report form was used to collect sociodemographic and clinical data. These included sex, date of birth, and classification of HIV disease, first-line ART and duration of treatment. Weight was measured using a Seca^®^weight scale, the height was measured by a wall mounted Seca^®^ 206 stadiometer. Body mass index (BMI) was calculated as weight (kg)/height (m^2^).Weight, height and BMI Z-scores were calculated using the British 1990 reference data [24]. Blood samples were collected to determine the viral load and CD4 cell counts. The CD4 T cell counts were determined using BD FACS Calibur and real time HIV-RNA levels (viral load) were measured with COBAS Ampliprep/Taqman 96 analyser with a detection range of 20 − 10,000,000 copies per ml.

###  Dual energy X-ray absorptiometry

Areal BMD measured in grams per square centimeter was determined using the DXA Hologic Discovery Wi DXA scanner with apex software version 3.1 (Hologic Bedford Inc. Bedford MA, USA). The output included bone area in square centimeters and bone mineral content (BMC) in grams. Two DXA technologists conducted the DXA scans.(coefficient of variation:0.66-1.02%) The method of BMD acquisition has been previously described [25]. Lumbar spine bone mineral apparent density(LS-BMAD) was calculated from the DXA measured LS BMD using Bachrach’s method [26]. Normative values for obtaining height adjusted Z- score were obtained from bone mineral density childhood study (BMDCS) reference data [27] since there are no African reference data. Low BMD was defined as a height adjusted Z-score of −2 or less as recommended by International Society for Clinical Densitometry(ISCD) [28] https://iscd.org/learn/official-positions/pediatric-positions/. The DXA scanner was calibrated daily using a spine phantom and auto air calibration for the whole body.

### Calcaneal quantitative ultra sound measurement

**Fig. 1:**
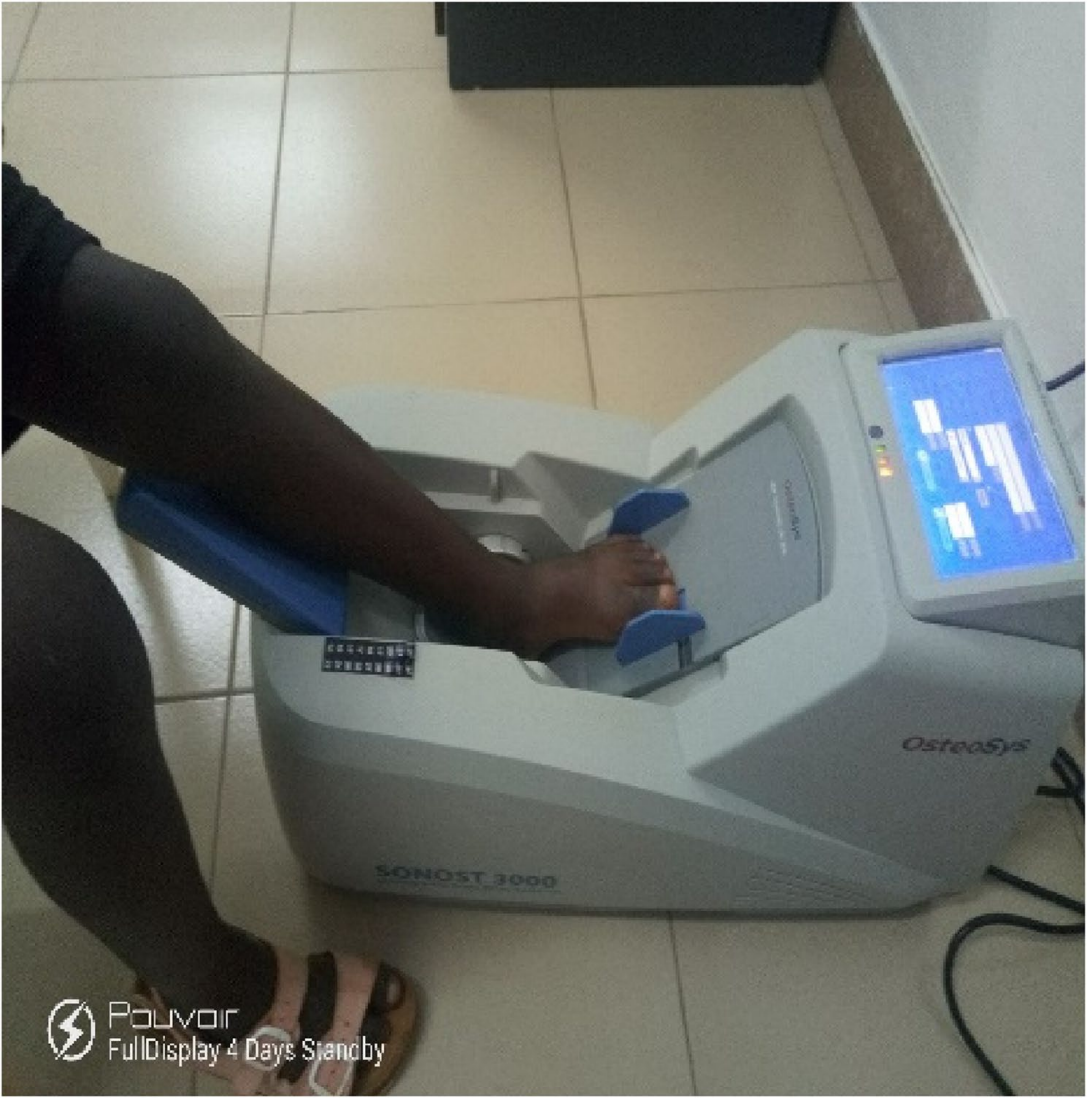
Calcaneal quantitative ultrasound device (*photo taken by the authors*)

Calcaneal QUS measurements were obtained by two trained operators (coefficient of variation: 2.72: SD = 4.02%) using SONOST 3000 (Osteosys. Seoul, South Korea) a calcaneal QUS device (Fig. 1) according to the manufacturer’s manual [29]. The readings were taken by placing the non-dominant foot into the calcaneal QUS machine at the calf support. The participant was asked to sit on a chair and remained still. Coupling gel was applied to the heel with the index and middle fingers. The foot was held in position by coupling pads and foot supporter. The QUS device automatically sends a signal in case of improper foot positioning prompting the study team to repeat the procedure. The speed of sound (SOS) measured in meters per second and broad band ultra sound attenuation (BUA)measured in decibel-Hertz (dB/MHz) were generated by the device on a print out. The device also generated other values namely, the bone quality index (BQI) which is equal to αSOS + βBUA and a BQI Z-score which is based on National Health and Nutrition Examination Survey (NHANES)reference data [29].

The procedure was repeated three times without moving the foot to obtain three readings per patient and the average was computed. The QUS and DXA measurements were obtained on the same day or within 2 weeks if the two procedures were not performed on the same day.

The QUS machine was calibrated daily using a phantom prior to taking the measurements according to the manufacturer’s manual [29] https://osteosys.com/en/product/sonost3000/.

### Statistical analysis

Descriptive characteristics of the study participants were summarized. The variables included age, sex, first line ART and duration, CD4 cell counts and percentage, viral load, BMI, and WHO disease stage. Medians and their corresponding interquartile ranges were obtained for continuous variables. Categorical variables were summarized using frequencies and percentages.

Height adjusted BMD Z-score were categorized into two. A Z-score less or equal to −2 defined low BMD while a Z-score above − 2 was normal BMD. The Z-scores were explored in assessing the ability of the QUS machine to detect low BMD determined by DXA machine.

The association between calcaneal QUS and DXA parameters was determined using Spearman correlations and P-values.

The receiver operating characteristic (ROC) curve was used to compare the ability of the calcaneal QUS with the DXA machine to identify children with low BMD.

Data were analyzed using Stata version 14.2 (Stata Corp).

## Results

The characteristics of 167 enrolled children are presented in Table 1. Their median age (IQR) was 9.4 years (6–12), BMI for age Z score (IQR) −0.94(−1.72, −0.39) and all the participants were black Africans. Most of the patients presented with a mild or asymptomatic WHO stage with 38.9% at stage I and 52.1% stage II. The median (IQR) CD4 cell count and viral load were 792 (550–1141) cells/µl and 17,252 (5369–69088) copies/ml respectively. All the patients had virologic failure as indicated in Table 1.

**Table 1:** Characteristics of study participants and summary of quantitative ultrasound and dual energy X-ray absorptiometry (DXA) measurements *N* = 167

Characteristic	Measure
Age: median (IQR)	9.4(6.0,12.0)
Male sex (n/%)	85.0 (50.9)
**Anthropometry**	
Weight-for-age Z-score (WAZ): median (IQR)	−1.29 (−2.16, −0.49)
Height-for-age Z-score (HAZ): median (IQR)	−1.03 (−1.56, 0.01)
BMI- for- age Z-score: median (IQR)	−0.94 (−1.72, −0.39)
**HIV characteristics**	
CD4 count (cells/ul): median (IQR)	792 (550, 1141)
CD4%, median (IQR)	31 (23, 37)
Viral load (copies/ml): median (IQR)	17,252(5369, 69088)
Time on first-line ART (years): median (IQR)	5.03(3.31,6.70)
**WHO Stage (n/N)**	
Stage 1	65(38.9)
Stage II	87(52.1)
Stage III	12(7.2)
Stage IV	3(1.8)
**Calcaneal QUS**:	**Median (IQR)**
SOS, m/s	1514.1(1507.8,1522.13)
BUA (dB/MHz)	76.0(64.2,87.9)
BQI	76.5(67.9,84.4)
BQI Z-score	−0.30(−0.70,0.17)
**DXA BMD**:	
**Lumbar spine**	
BMAD g/cm^3^	0.091(0.082,0.102)
BMD g/cm^2^	0.536(0.483–0.644)
BMD Z-score	−1.7 (−2.50, −1.00)
Height adjusted BMD Z-score	−0.99(−1.43, −0.34)
Low lumbar BMD n/N (%)	21/159 (13.2)
**TBLH**	
BMD g/cm^2^	0.650 (0.554, 0.731)
BMD Z-scores	−2.0(−2.7, −1.3)
Height adjusted BMD Z-scores	−1.37 (−1.87, −0.81)
Low TBLH BMD n/N (%)	28/159(17.6)

*IQR *Interquartile range, *BMI *Body mass index, *SOS *Speed of sound, *BUA *Broad band ultra sound attenuation, *QUS *Quantitative ultra sound, *BQI *Bone quality index, *BMD *Bone mineral density, *BMAD *Bone mineral apparent density, *TBLH *Total body less head

The median (IQR) duration on first-line ART in years was 5.03 (3.31–6.70). The median SOS (IQR) was 1514.1(1507.8-1522.1). Median BUA (IQR) was 76(64.2–87.9). The mean BQI (IQR) was 76.5(67.9–84.4).

The median (IQR) total body less head (TBLH) BMD was 0.650 g/cm^2^ (0.554–0.731) which was comparable with the median (IQR) lumbar BMD of 0.536 g/cm^2^ (0.483–0.644).

Correlations between the DXA and QUS parameters are shown in Table 2. Among the QUS measurements, a moderate correlation was observed between DXA measured value and the BUA ranging from 0.47(TBLH BMC) to 0.50 (TBLH BMD). The BQI was moderately correlated with the TBLH BMC (*R* = 0.46, *P* < 0.001), TBLH BMD (*R* = 0.46, *P* < 0.001) and LS BMC (*R* = 0.41, *P* < 0.001).

**Table 2:** Correlations between DXA and calcaneal quantitative ultra sound parameters (p-values)

	Calcaneal QUS			
	SOS	BUA	BQI	BQI Z- score
	r_s_ (p-value)	r_s_(p-value)	r_s_(p-value)	r_s_(p-value)
**Lumbar spine**				
BMD	0.05(0.51)	0.05(0.52)	0.07(0.40)	−0.09(0.27)
BMAD	0.19(0.01)	0.28(< 0.001)	0.29(< 0.001)	0.07(0.35)
BMC	0.31(< 0.001)	0.43(< 0.001)	0.41(< 0.001)	0.11(0.15)
BMD Z-score	0.07(0.43)	−0.00(0.98)	0.05(0.63)	0.23(0.01)
**TBLH**				
BMD	0.35(< 0.001)	0.50(< 0.001)	0.46(< 0.001)	0.16(0.04)
BMC	0.34(< 0.001)	0.47(< 0.001)	0.46(0.001)	0.13(0.10)
BMD Z- score	0.22 (0.015)	0.10(0.26)	0.17(0.06)	0.30(0.001)

*QUS *Quantitative ultra sound, *SOS *Speed of sound, *BUA *Broadband ultra sound attenuation, *BQI *Bone quality index, *TBLH *Total body less head, *BMC *Bone mineral content, *BMD *Bone mineral density, *BMAD *Bone mineral apparent density, r_s_spearman rho

There was weak correlation between SOS and DXA parameters. The correlation between SOS and TBLH BMD was weak (*R* = 0.35, *P* < 0.001), so was LS BMC (*R* = 0.31, *P* < 0.001) and LSBMAD (*R* = 0.19, *P* = 0.01).

QUS Z-scores were weakly correlated with TBLH BMD and LSBMD Z-scores (*R* = 0.30, *P* < 0.01) and (*R* = 0.24, *P* < 0.01) respectively.

**Fig. 2:**
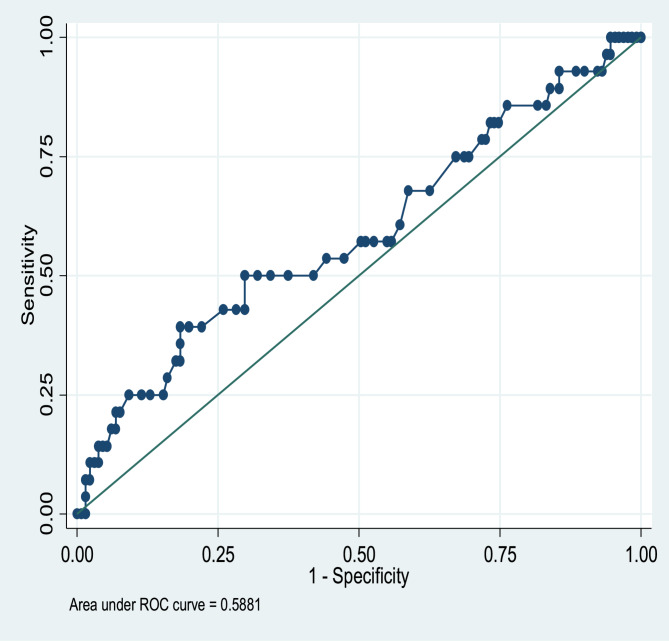
Receiver operating characteristic (ROC) curve for QUS in detecting low BMD defined as DXA TBLH BMD or LSBMD height adjusted Z-score ≤ 2

The ROC curve revealed that QUS was a weak predictor of DXA Z-score equal to or less than − 2 (area under the ROC curve = 0.59, Fig. 2).

Using a DXA TBLH height -adjusted Z-score of ≤ −2 as a reference for diagnosing low BMD, the QUS sensitivity, specificity, positive predictive value (PPV) and negative predictive values (NPV) are presented in Table 3. The sensitivity was 50% for the QUS while the specificity was 83.7%. PPV was 10.7% and NPV was approximately 98%. The QUS missed the diagnosis of 50% of participants with low TBLH BMD who were diagnosed by DXA.

**Table 3:** The ability of QUS Z-score at different cut points to determine DXA determined low BMD

BQI Z-score cut points	Sensitivity	specificity	PPV	NPV
0.0	18.06	100	100	03.05
−0.1	18.95	84.38	64.29	41.22
−0.3	19.51	84.42	57.14	49.62
−0.73	29.73	86.07	39.29	80.15
−1.00	31.82	84.67	25.00	88.55
−1.30	44.44	84.00	14.29	96.18
−1.40	50	83.66	10.71	97.71
−1.46	50	83.23	7.14	98.47
−1.48	50	83.23	7.14	98.47
−1.50	50	83.23	7.14	98.47
−1.52	50	83.23	7.14	98.47
−1.54	33.33	82.69	3.57	98.47
−1.58	33.33	82.69	3.57	98.47
−1.70	33.30	82.69	3.57	98.47
−1.80	0.0	82.17	0.00	98.47

*PPV *Positive predictive value, *NPV *Negative predictive value, *BQI *Bone quality index

## Discussion

Our main findings were that the correlation between DXA and calcaneal QUS values ranged from weak to moderate. Calcaneal QUS may not accurately determine low BMD in CLWH. Our data are consistent with other paediatric studies comparing QUS and DXA scanner. In a cross sectional study in England in a population of 107 healthy adolescents, Costoso et al. compared QUS with DXA, and found a moderate correlation between BUA and DXA parameters namely TBLH and LS BMC and a moderate correlation with the SOS.(*r* = 0.54 and 0.50 respectively) [30]. These findings are similar to our findings in which the SOS was weakly correlated with the DXA measurements (*r* = 0.31), while the BUA was moderately correlated with the DXA measurements. These findings are also consistent with those of an adult study among adults in the Vietnam osteoporosis study in which BUA was modestly correlated with LSBMD(*r* = 0.35) hence QUS is less likely to be a useful tool [31].

Jackson et al. investigated the correlation between the two devices in 80 CLWH in South Africa and found a moderate correlation between the QUS BUA and whole-body BMC as well as BMD obtained by DXA which is similar to our study findings. However, there was no correlation between the SOS and DXA parameters [19]. Similarly, Weeks et al. reported positive but moderate correlations between BUA and DXA TBLH measurements in healthy Australian children (*r* = 0.46 to 0.54) [32]. Similarly, the BQI correlated with the DXA TBLH BMC and BMD in healthy Chinese children [33].

Bak-Drabik et al. investigated the correlation between DXA and QUS among 51 children with inflammatory bowel disease in Poland and reported a strong correlation between the QUS SOS and TBLH BMD but a weak correlation with Z-scores [34]. In contrast to our findings, the SOS was weakly correlated with all the DXA parameters. A possible explanation could be differences in methods used to determine the SOS. The Amplitude dependent SOS was used but we used an SOS that did not vary with intensity. Secondly there was a difference in body parts that were used to assess for SOS score. We used the calcaneal bone in our study while the authors measured the hand phalanges; hence comparison of the two groups is challenging. The bone properties that are reflected by SOS which is microarchitecture are different from the properties that are captured by the DXA machine hence the weak correlation. There was a weak correlation between Z scores of the DXA and QUS in our study, which was similar to the findings of other studies [34, 35]. A possible explanation for this finding is that the two devices measure different bone properties. The BMD obtained from DXA measurement is the bone mineral content while the QUS provides information about the bone microarchitecture that contributes to bone strength. Secondly the different body sites, the calcaneal bone and the TBLH were assessed and compared. The calcaneal bone is largely trabecular while TBLH DXA assessments mainly focus on cortical bone [36].

Ethnicity may play a role in shaping the correlation between BMD and bone quality indices observed in this study. Previous literature has reported ethnic differences in bone health, with the black population demonstrating higher trabecular and cortical BMD and bone quality compared with the White and Asian populations [26, 37]. These differences may influence the variation in both the absolute values and the relationship between DXA and QUS parameters. In our study, the BMD Z-scores were based on a reference population of the Black population on the BMDCS study, whereas the BQI Z-scores were derived from NHANES data with a population that maybe exposed to distinct environmental and possibly lifestyle factors. Although both populations were Black, differences in genetics, nutrition, physical activity, sunlight exposure, and socioeconomic conditions may contribute to variation in BMD measurements. These factors may affect the strength and direction of correlation between BMD and BQI measurements. Future research should aim at using normative values that can account for ethnic, regional and environmental factors to enhance the comparison of the measurements.

The sensitivity of QUS was low in our study, missing 50% of participants with low BMD therefore QUS is considered a poor diagnostic tool. These findings are similar to those from the Biobank cohort from the UK where QUS identified 0.34 to 4.9% of participants with osteoporosis and had low to modest correlations (*r* = 0.29 to 0.44) [38].

The strengths of the study are, that BMD was adjusted to height as recommended by the ISCD since bone size compromises BMD. Secondly, this is among the largest documented comparisons of DXA and QUS for determining BMD among CLWH with viral non-suppression. Previous studies had small sample sizes and a few focused on CLWH [39].

Limitations include, a foot size less than 19 cm tends to give abnormally high SOS values [17]. In this study foot size was not measured hence participants with foot size less than 19 cm were not excluded from the analysis, though none of the SOS readings were above 1620 m/s. There were no local QUS reference curves, the reference data for DXA generated BMD were from the BMDCS study while those for the QUS BQI- Z score were from the NHANES.

Our findings may not be generalizable given that there are many types of QUS devices with variations.

The DXA and QUS have their limitations as well. Much as height adjustment enhances the accuracy of the DXA output, it does not completely remove its dependency on a two-dimensional imaging that cannot capture the three-dimensional complexity of bone structure. The DXA only reports the bone mineral content excluding the microarchitecture [12]. The QUS also has its shortcomings in that it only captures peripheral bone measurements thus excluding the axial skeleton. This may lead to missing low BMD in the axial skeleton.

The calcaneal bone is composed of trabecular bone which is more metabolically active and sensitive to change compared with the cortical bone. Anatomical site matched measurements may improve the correlation between the DXA and QUS devices.

This was a single site study. Cortical and trabecular bone may vary across different sites.

In conclusion, among CLWH, there was a weak to moderate correlation between DXA and QUS parameters. QUS may not accurately determine BMD where a DXA scan is unavailable. The outcomes of QUS are varied and difficult to compare given the variation in devices used. Longitudinal data from the CHAPAS-4 trial are still pending to further assess longitudinal observations and the need to explore cost effective alternatives to DXA the gold standard still stands.

## Data Availability

The datasets generated and/or analyzed during the current study are not publicly available due [to the Joint Clinical Research Centre institutional data policy] but are available from the corresponding author on reasonable request.

## Abbreviations

ART: Anti- retroviral therapy
BMD: Bone mineral density
BMC: Bone mineral content
BMAD: Bone mineral apparent density
BQI: Bone quality index
BUA: Broad band ultra sound attenuation
CLWH: Children living with HIV
CHAPAS-4: Children with HIV in Africa Pharmaco-kinetics and Adherence of Simple anti-retroviral regimens
DXA: Dual energy X-ray absorptiometry (DXA)
ISCD: International society of clinical densitometry
IQR: Inter quartile range
JCRC: Joint clinical Research Centre
LS: Lumbar Spine
PLWH: People living with HIV
QUS: Quantitative ultra sound
RLS: Resource limited setting
SOS: Speed of sound
TBLH: Total body less head
